# Long-term clinical prognosis of patients with gliomas infiltrating corticospinal tract on DTI tractography

**DOI:** 10.1186/s41016-025-00412-8

**Published:** 2025-10-24

**Authors:** Xijie Wang, Zhentao Zuo, Songlin Yu, Song Lin, Tao Yu

**Affiliations:** 1 Beijing Neurosurgical Institute, Beijing, 100070 China; 2 Department of Neurosurgery, Beijing Tiantan Hospital, Capital Medical University, Beijing, 100070 China; 3 National Clinical Research Center for Neurological Diseases, Beijing, 100070 China; 4 State Key Laboratory of Brain and Cognitive Science, Institute of Biophysics, Chinese Academy of Sciences, Beijing, 100101 China

## Abstract

**Background:**

The infiltration of the corticospinal tract (CST) in patients with gliomas may lead to more postoperative paralysis and worse survival than others. The aim of this study is to investigate the clinical outcomes and propose the surgical strategy for these patients.

**Methods:**

We retrospectively identified 101 patients with CST infiltrated by cerebral gliomas on preoperative DTI tractography. Surgical, neurologic, and oncological outcomes were assessed on long-term follow-up.

**Results:**

Forty-eight (47.5%) patients harbored grade II gliomas, 26 (25.7%) had grade III gliomas, and 27 (26.7%) had grade IV gliomas. Gross-total resection (GTR) or subtotal resection (STR) was achieved in 67.3% of patients, and partial resection (PR) was achieved in 32.7% of patients. Large tumors (≥ 24.5 ml) and low-grade gliomas (LGGs) were independent prognostic factors for partial resection. Patients with high-grade gliomas (HGGs) and pre-operative motor deficit had a higher risk for permanent paralysis. Thirty-three of 101 patients (32.7%) had long-term paralysis, and 7 patients (6.9%) suffered from severe paralysis. The median PFS and OS were 12 months and 24 months in grade IV gliomas. In multivariate analysis using the Cox model, low tumor grade and IDH1 mutation were independent factors for longer PFS, and low tumor grade was an independent factor for longer OS.

**Conclusion:**

Preoperative DTI tractography is a valuable tool for determining the extent of CST involvement in patients with gliomas. The risk of postoperative paralysis is extremely high; therefore, careful and conservative resection should be performed to preserve motor function. Despite this challenge, patients can still achieve positive oncological outcomes with standard adjuvant therapy after surgery.

## Background

Cerebral gliomas are highly invasive neoplasms, often located in or adjacent to eloquent areas. Treatment of gliomas poses a challenge for neurosurgeons, especially when they invade the primary motor cortex (M1) and corticospinal tract (CST) [1–3]. There is increasing evidence that extensive surgical resection of gliomas is associated with prolonged survival, but this must be balanced with the risk of irreversible neurological deficits [1–4]. Postoperative permanent motor deficits can greatly impact quality of life or hinder the administration of adjuvant treatments [5]. The preoperative noninvasive methods of motor mapping such as fMRI and DTI, are commonly used for predicting surgical risks, planning surgical approaches, and evaluating the resectability of the tumor [1, 6–8].

Intraoperative direct electrical stimulation (DES) is considered the gold standard, helping neurosurgeons to spare functional tissues from damage [1, 5, 9–11]. In recent years, DTI of white matter tracts was demonstrated to concur well with intraoperative mapping [9]. Furthermore, DTI may be better poised to inform patient selection, tumor grading and surgical planning [7, 12, 13]. Several studies have investigated the predictive value of DTI, which evaluates white matter integrity by visualizing water diffusion characteristics [14, 15]. CST involvement on DTI tractography has been found to correlate with postoperative functional outcomes [16]. The extent of CST involvement can predict the extent of resection (EOR) [7]. However, the predictive value of motor cortex or CST infiltration for tumor recurrence or patient survival in motor-eloquent area gliomas remains unclear [10].

In this study, we retrospectively reviewed 101 patients with gliomas infiltrating CST. All patients underwent preoperative DTI for evaluating surgical risk and planning the approach. Surgical resections were guided by intraoperative brain mapping. Motor deficits, EOR, tumor recurrence, and patient survival were reviewed. The treatment strategy for gliomas in highly motor-eloquent areas will also be discussed.

## Methods

### Patient population

We analyzed data from a consecutive series of 400 cases pathologically diagnosed with gliomas and who underwent preoperative DTI at the Neurosurgery Department IV of Beijing Tiantan Hospital between January 2014 and January 2019. All patients provided informed consent approved by the Ethics Committees of Beijing Tiantan Hospital (KY2023-156-03). Demographic and clinical data of the patients are presented in Table 1. The tissue sections of all patients were reviewed by senior neuropathologists who assigned diagnoses based on the 2016 WHO classification scheme [17]. Patients received treatment according to the latest National Comprehensive Cancer Network guidelines, including resection, postoperative temozolomide (TMZ)-based chemotherapy, and radiotherapy. The postoperative adjuvant therapies for 48 patients with LGGs included: radiotherapy alone for 4 patients, chemotherapy alone for 12 patients, concurrent radio-chemotherapy for 10 patients, and no adjuvant therapy for 22 patients. For the 53 patients with high-grade gliomas: radiotherapy alone for 3 patients, chemotherapy alone for 6 patients, concurrent radio-chemotherapy for 33 patients, and no adjuvant therapy for 11 patients. Details of the treatment protocol have been described in a previous study [17]. Preoperative and postoperative neurological evaluations were conducted for all patients.

**Table 1:** Patient demographic and clinical characteristics

Characteristic	All patients (*n* = 101)
Gender (no. of patients)	
Male	57
Female	44
Age	
Mean±SD	40.08 ± 12.01
Range	13~68
Follow-up (months)	
Median	51
Range	3–104
Primary presenting symptom (%)	
Seizure	50 (49.5%)
Motor weakness	22(21.8%)
Headache	16 (15.8%)
Sensory deficit	9 (8.9%)
Language disturbance	8 (7.9%)
Tumor grade	
II	48 (47.5%)
III	26 (25.7%)
IV	27 (26.7%)
Preop tumor vol (ml)	
Mean±SD	49.5 ± 45.4
Range	1.6~196.0
Extent of resection	
Grand total	39(38.6%)
Subtotal	29(28.7%)
Partial	33(32.7%)

### Imaging protocol

MRI data were performed at a Siemens Verio 3.0 Tesla MRI scanner (Siemens Healthineers, Erlangen, Germany). A 12-channel head coil was used for the reception of MR signal. The structural images were obtained in a sagittal orientation employing a magnetization prepared rapid gradient echo (MPRAGE) sequence over the whole brain: 176 slices, TI/TR/TE = 900/1900/2.52 ms, flip angle: 8°, slice thickness 1.0 mm, field of view = 250 × 250 mm^2^, matrix = 256 × 256. DTI data were obtained using a single-shot echo planar imaging sequence (TR/TE 11000/95 ms). Diffusion gradients were applied along 30 directions, using a b-value of 0 and 1000 s/mm^2^. A field of view (FOV) of 256 × 256 mm^2^ and a data matrix of 128 × 128 were used, leading to isotropic voxel dimensions (2.0 × 2.0 × 2.0 mm^3^). 65 slices were obtained, with a thickness of 2 mm and no gap.

### Image analysis

DTI Tractography was performed by Z.Z. Images were analyzed using FMRIB Software Library (FSL) [18]. Eddy current correction was applied using FSL. Brain mask was extracted using BET. Autoptx was used to track the CST automatically [19]. The CST was isolated by drawing an “OR” region of interest around the CST in the anterior part of the brain stem) and an “AND” region of interest around the corona radiata in the direction-coded color axial sections. Finally, reconstructed white matter tracts were superimposed on volumetric postcontrast T1-weighted or FLAIR images. This allowed comparison of the trajectories of the tracts in the involved hemisphere with those of the contralateral unaffected hemisphere and evaluation of the anatomical relationship between the tract and the tumor mass and the effect exerted by the tumor on the tract of interest.

Involvement of CST was considered as infiltration if the following criteria were met: (1) the border of CST was closely adhered to the tumor with significant deformation. (2) The CST exhibited defects or discontinuity compared to the control side. (3) CST partially or entirely overlapped with the tumor or the peritumoral edema. The involvement of CST and its correlation with the glioma were assessed by Z.T.Z. and T.Y. For a few cases with discrepancies, the research team discussed to make sure the infiltration of CST.

### Surgery and perioperative evaluation

Muscle strength was evaluated and graded according to the Medical Research Council, UK. All patients underwent tumor resection by senior author S.L. under general anesthesia. Direct cortical and subcortical electrical stimulation was performed on each patient to determine the resection limit based on the identification of cortical or subcortical motor tracts. Patients were assessed on preoperative day 1, day of discharge, and during long-term follow-up for motor function evaluation.

Extent of resection (EOR) was assessed by calculating the volume of hyperintense signal on FLAIR sequence within 3 days post-surgery. Tumors were manually segmented across all slices, and the tumor volume was calculated using MRIcro. The EOR was classified as gross total resection (GTR) if the tumor was resected for ≥ 98% of volume; subtotal resection (STR) if the tumor was removed by 85–98%; and partial resection (PR) if the resection degree was less than 85%.

### Follow-up

Survival data, except for 4 cases, were primarily obtained during clinic visits or routine phone interviews conducted at 3-month intervals, or 1 month if necessary. All patients were followed until December 2024 or until death. Duration of survival was defined as the period from the index operation to death or last follow-up. At the time of data analysis, the median follow-up for the cohort was 51 months (range 3–104 months), with 32 patients having deceased.

### Statistical methods

Primary analyses were conducted using SPSS software (version 19.0, IBM Corp.). Chi-square and univariate logistic regression analyses were used to identify associations between categorical variables. Multivariate logistic regression analysis was performed to assess significant independent factors related to motor deficit and extent of resection. Kaplan-Meier analysis was utilized to calculate survival rates and the log-rank test was employed for univariate comparisons to investigate the association between parameters and progression-free survival (PFS) and overall survival (OS). A Cox proportional hazards model was utilized for multivariate analysis, including variables with a *p*-value < 0.05 from the univariate analysis. All tests were 2-sided, and a *p*-value < 0.05 was considered statistically significant.

## Results

### Patient population

Infiltration of the CST was identified in 101 patients, consisting of 57 males and 44 females with a mean age of 40.08 years (range, 13–68 years). Descriptive characteristics of this cohort are summarized in Table 1. The majority of patients (94 out of 101) were right-handed. Clinical history revealed that 50 patients had a history of seizures, followed by 22 focal motor weakness, 16 headache, 9 sensory deficit, and 8 language disturbance. There were no cases of operative mortality. Histological classification based on the World Health Organization brain tumor classification revealed that 48 patients had grade II glioma, 26 patients had grade III glioma, and 27 patients had grade IV glioma.

### Extent of resection

The mean extent of resection was 89.0% ± 12.3% (range, 55–100%). In this cohort, gross total resection (GTR) was achieved in 39 patients, subtotal resection (STR) in 29 patients, and partial resection (PR) in 33 patients. The average resection rates for WHO grade II, III, and IV gliomas were 86.7% ± 13.4%, 88.7% ± 12.6%, and 93.5% ± 8.4%, respectively.

**Fig. 1:**
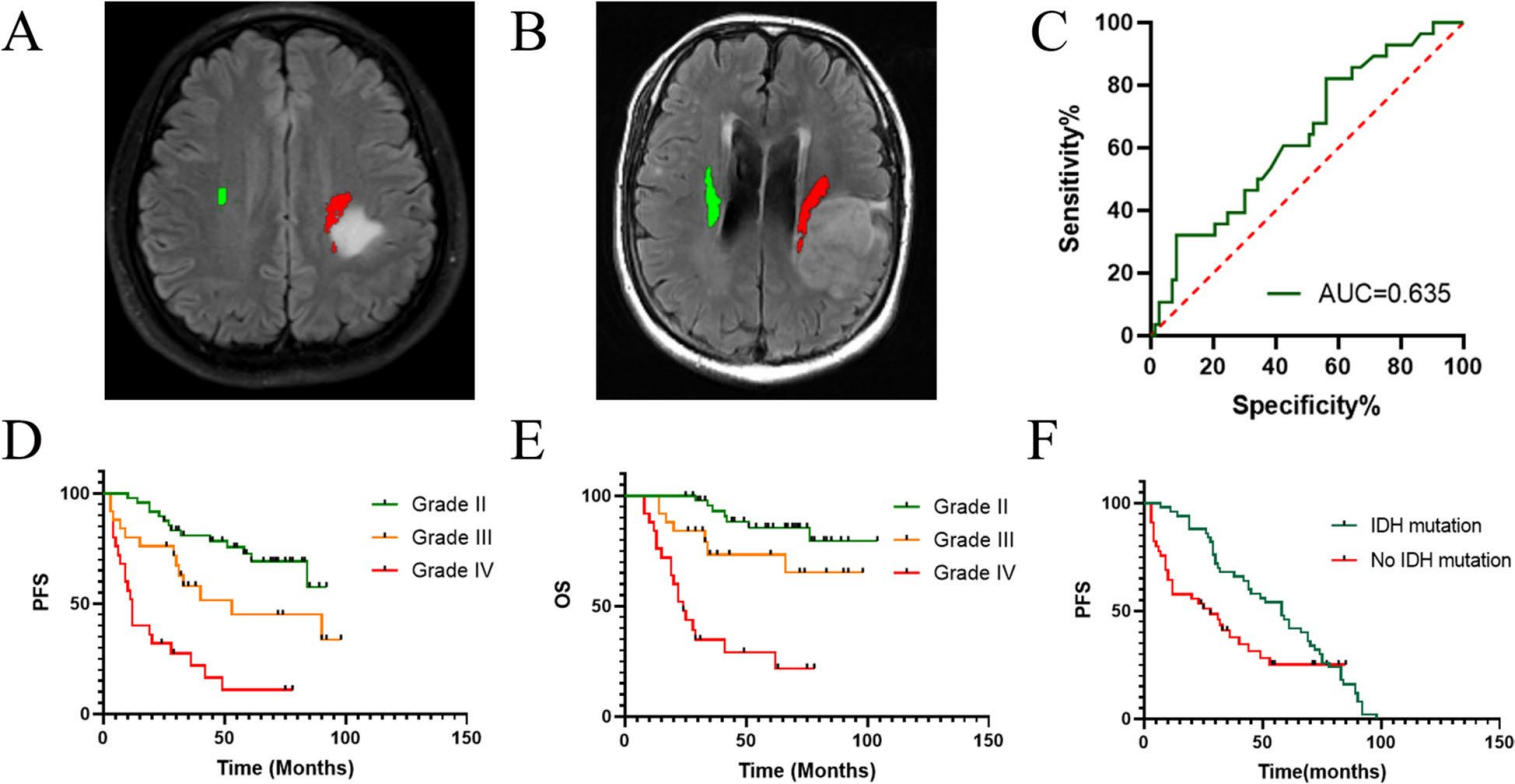
CST infiltrated by grade II glioma (**A**) and grade IV glioma (**B**). Receiver operating characteristic (ROC) curve analysis for tumor volume in predicting PR (**C**). Kaplan–Meier curves of PFS (**D**) and OS (**E**) of all patients of tumor grade and PFS of patients of IDH mutation (**F**)

GTR or STR were achieved in 60.4% of patients with grade II gliomas, 73.1% in grade III gliomas, and 92.6% in grade IV gliomas. Receiver operating characteristic (ROC) curve analysis for tumor volume in predicting PR showed an area under the curve (AUC) of 0.635. The optimal cut-off value for tumor volume was 24.5 ml, with a sensitivity of 82.1% and specificity of 56.2% (Fig. 1). Univariate logistic regression indicated that PR was associated with tumor volume (≥ 24.5 ml) (*p* = 0.022) and low-grade gliomas (LGGs) (*p* = 0.030) (Table 2). Multivariate logistic regression further confirmed that PR was significantly associated with LGGs (*p* = 0.006) and large tumor volume (≥ 24.5 ml) (*p* = 0.013) (Table 2).

**Table 2:** Chi-square and multivariate analysis of extent of resection

		Chi-square				Multivariate	
Variables		GTR or STR	PR	*p* value	Variables	HR of PR (95% CI)	*p* value
Tumor grade	LGGs	29	19	0.030	HGGs vs LGGs	3.839(1.458–10.112.458.112)	0.006
	HGGs	44	9				
Tumor volume	≥ 24.5 ml	42	23	0.022	< 24.5 ml vs ≥ 24.5 ml	0.240(0.078–0.738.078.738)	0.013
	< 24.5 ml	31	5				
Preoperative paralysis	Yes	19	3	0.310			
	No	54	25				

*GTR* gross total resection, *STR* subtotal resection, *PR* partical resection

### Functional outcomes

Long-term language deficits were reported in 4.0% (4 out of 101) of patients, while permanent motor deficits (≥ 6 months follow-up) were observed in 33 out of 101 patients (32.7%) (Table 3). Among these 33 patients, 22 had preoperative motor deficits. Severe motor deficits (grade 3 muscle strength) were noted in 7 patients (6.9%), and the remaining 26 patients (25.7%) with motor deficits exhibited grade 4 muscle strength or mild motor deficits. Univariate logistic regression indicated that postoperative paralysis was associated with EOR (*p* = 0.060), preoperative paralysis (*p *= 0.000) and HGGs (*p* = 0.000) (Table 3). Multivariate logistic regression analysis revealed that patients were more likely to experience permanent motor deficits if they had HGGs (*p* = 0.001) or preoperative motor deficits (*p *= 0.003) (Table 4).

**Table 3:** Univariate analysis of surgical outcomes

		Without paralysis (*n *= 68)	Long-term paralysis (*n* = 33)	*p* value
Tumor volume	ml	39.51	37.38	1.000
EOR	GTR or STR	45	28	0.060
	PR	23	5	
Pathology	LGG	43	5	0.000
	HGG	25	28	
Preoperative motor function	W/o limb weakness	63	16	0.000
	With limb weakness	5	17

**Table 4:** Multivariate analysis of risk of postoperative paralysis

Variable	PFS	
	HR(95%CI)	*p* value
EOR (GTR or STR vs. PR)	0.654 (0.179–2.381)	0.519
Pathology (HGGs vs LGGs)	6.693(2.078–21.5554)	0.001
Pre-op paralysis (yes vs no)	9.372(2.738–32.084)	0.003

### Recurrence analysis

Out of the 101 patients, 48 experienced tumor recurrences over a span of 3–98 months. Among the 14 patients with LGGs, tumor recurrence occurred over 10–84 months. For patients with HGGs, 34 individuals had tumor recurrence within 3–90 months. The median progression-free survival (PFS) was 53.0 months for grade III glioma patients and 12.0 months for grade IV glioma patients. Univariate analysis using the Cox model identified postoperative paralysis, age, tumor grade, loss of heterozygosity (LOH) 1p19q, and IDH1 mutation as prognostic factors (Table 5). Multivariate analysis using the Cox model indicated that high tumor grade (*p *= 0.017) and IDH1 mutation (*p *= 0.004) independently increased the risk of recurrence (Fig. 1).

**Table 5:** Progression-free survival analysis of the prognostic factors

Variable	PFS		Multivariate	
	HR(95%CI)	*p* value	HR(95%CI)	*p* value
Postoperative paralysis (yes vs. no)	0.388 (0.219–0.690)	0.001	0.601(0.309–1.166)	0.132
Age (< 60 years vs. > 60)	2.178 (1.142–4.153)	0.018	1.119(0.549–2.281)	0.757
EOR (PR vs. GTR or STR)	1.143 (0.840–1.556)	0.395		
Tumor grade	2.667 (1.865–3.812)	0.000	1.685(1.099–2.582)	0.017
LOH 1p19q (yes vs. no)	0.217 (0.077–0.608)	0.004	0.373(0.125–1.107)	0.076
IDH1 mutation (yes vs. no)	0.295 (0.159–0.546)	0.000	0.347(0.169–0.714)	0.004

### Survival analysis

Within 8 to 76 months, 32 out of 101 patients (31.9%) succumbed to tumor recurrence, including 7 patients with LGGs and 25 patients with HGGs. The median overall survival (OS) was 24.0 months for patients with grade IV glioma and was not reached for patients with other pathological types. Univariate analysis using the Cox model revealed that postoperative paralysis, age ≥ 60, HGGs, loss of heterozygosity (LOH) 1p19q, and IDH1 mutation were significant prognostic factors for OS (Table 6). Multivariate analysis using the Cox model demonstrated that high tumor grade was an independent prognostic marker for OS (*p *= 0.008) (Table 6) (Fig. 1).

**Table 6:** Overall survival analysis of the prognostic factors

Variable	OS		Multivariate	
	HR(95%CI)	*p* value	HR(95%CI)	*p* value
Postoperative paralysis (yes vs. no)	0.317 (0.157–0.637)	0.001	0.691(0.297–1.608)	0.391
Age (< 60 years vs. ≥ 60)	2.520 (1.187–5.353)	0.016	1.413(0.600–3.328)	0.429
EOR (PR vs. GTR or STR)	1.446 (0.684–3.056)	0.334		
Tumor grade	3.416 (2.172–5.374)	0.000	2.073(1.210–3.551)	0.008
LOH 1p19q (yes vs. no)	0.027 (0.001–0.757)	0.034	0.000(0.000–4.779)	0.944
IDH1 mutation(yes vs. no)	0.008 (0.176–0.767)	0.000	0.600(0.251–1.437)	0.252

## Discussion

The present retrospective study offers unique insights into the long-term functional and oncological outcomes of patients with gliomas in highly-motor eloquent areas. The infiltration of the CST identified through DTI tractography served as a crucial indicator for distinguishing patients’ outcomes. The findings indicated that CST infiltration significantly elevated the risk of postoperative paralysis, particularly in patients with HGGs.

In recent years, preoperative DTI tractography has become increasingly utilized to predict surgical outcomes for brain tumors [7, 20, 21]. For gliomas situated in or near motor eloquent regions, some cases were deemed “unresectable” due to the high risk of iatrogenic injury during resection [5]. Studies, such as by Sollmann et al., have defined cut-off values for lesion-to-tract distances (LTDs) to assess the risk of surgery-related paresis, with LTD-CST values of ≤ 8–12 mm [22]. Surgical resection becomes more challenging when LTD is 0 or CST is dislocated. The extent of CST involvement can be categorized into normal form, displacement, and infiltration or disruption. This grading system for CST involvement aids neurosurgeons in determining the feasibility of tumor resection and predicting patient functional outcomes [7, 23]. Quantitative tractography measurements has been reported to be a useful approach for predicting clinical outcomes in patients with LGGs involving the left temporo-insular cortex [24–27].

Previous research has shown that increasing tumor volume poses challenges for achieving total resection, particularly in eloquent areas [28]. Study by Castellano et al. have indicated that the presence of CST infiltration or displacement predicts a lower likelihood of total resection, especially for tumors with a preoperative volume < 100 ml [7]. Our study also observed a higher resection rate for tumors with volumes < 24.5 ml, potentially attributed to the greater extent of involvement in our current study. But the AUC was 0.635, so the result has weak significance and lacks practical significance. Additionally, a lower resection rate was noted in LGGs cases. In LGGs, which are associated with longer survival, preserving quality of life is paramount, so surgeons may exercise greater caution. Conversely, in aggressive HGGs, maximizing resection is often necessary to prolong survival. Aggressive resection in HGG patients may resulted in poorer functional outcomes.

During surgical resection, when using intraoperative DES to detect CST on the resection interface, the resection can typically be limited to the area near the tumor-CST boundary, reducing the risk of accidental injury to the intact CST [3, 9]. However, for patients with CST infiltration, the resection may need to be halted before the tumor is completely removed [3]. Despite strict guidance from DES in our study, the postoperative motor functional outcomes were poor, with 32.7% of patients experiencing permanent motor deficits, including 7 severe cases. Many experts advocate for conservative surgical approaches to prevent inadvertent motor tract injuries [29]. It is crucial to carefully weigh the benefits and risks of aggressive resection, considering the importance of preserving the CST for motor function and self-care ability. Skilled neurosurgeons and proficient use of intraoperative DES and DES-based subcortical mapping are crucial for achieving this balance and ensuring optimal functional outcomes [1, 3, 30].

In this study, it was observed that patients with HGG had a higher incidence of permanent motor deficit compared to those with LGGs [31, 32]. The presence of unclear boundaries in gliomas, especially HGGs, can make it challenging to determine the appropriate resection interface. In HGGs, once the CST is infiltrated by tumor cells or located within the peri-tumor edema, achieving hemostasis is very challenging. During the hemostatic process in this area, the risk of unintentionally damaging the CST significantly increases. Therefore, if direct electrical stimulation elicits a response, the distance from the excision boundary to the CST may only be 1~2 cm. A safer approach at this point is to initiate hemostasis rather than continue tumor resection. The theory of motor function remodeling also supports this practice. According to Duffau’s theory, the rapid growth of HGGs not only increases involvement of the CST but also limits motor plasticity, as functional motor fibers may intertwine with malignant tumor tissues [33]. Removing these fibers can lead to paralysis and hinder recovery opportunities, emphasizing the need for more conservative surgical approaches for HGGs.

While increased resection of gliomas may reduce tumor burden for postoperative chemotherapy and radiotherapy, potentially prolonging patient survival, it is essential to consider factors like deep white matter invasion, which can predict disease progression in patients with grade IV glioma [5, 34]. Although radical resection has been linked to improved outcomes for both LGG and HGG, pursuing GTR may not always be beneficial for improving PFS and OS [1, 9, 21]. The study’s results indicated that patients with grade IV glioma had PFS and OS rates of 12 months and 24 months, respectively, comparable to outcomes in the majority of cases in the ward. While aggressive resection may impair motor function without necessarily improving oncological outcomes, further research is warranted to explore strategies for enhancing prognosis in these patients.

High-grade tumor had worse PFS and OS, as shown in Tables 5, 6, and Fig. 1. While IDH1 mutation appears to influence PFS but not OS. We infer that it was because patients had different treatments after tumor recurrence, such as re-operation, radiotherapy, chemotherapy, or conservative treatment. Therefore, our data may not truly reflect the impact of IDH mutation on OS.

This study has several limitations that should be acknowledged. Firstly, the lack of standardized DTI tractography algorithms in the study may have impacted the results and conclusions due to variations in reconstruction methods. The high morbidity rate suggests our DTI results may underestimate actual tumor infiltration into motor tracts. Because we did not use qualitative analysis, interobserver reliability cannot be ignored in evaluation of CST involvement. Secondly, being a retrospective study from a single institution, there may be significant differences in surgical strategies among different authors. Future studies with multicenter participation are needed to validate the findings. Thirdly, the overall sensitivity of DTI models in detecting all motor pathways is limited, highlighting the need for complementary techniques such as preoperative navigated transcranial magnetic stimulation (nTMS) to accurately locate motor areas and ensure safe resection of motor cortex tumors [14, 35, 36].

## Conclusions

Preoperative DTI tractography is a valuable tool for determining the extent of CST involvement in patients with gliomas. CST infiltration increases the risk of postoperative paralysis, especially in cases of HGGs. Unforeseen damage to the CST may contribute to the higher incidence of postoperative hemiplegia. Therefore, a careful and conservative surgical resection, combining with intraoperative electrophysiological monitoring, is essential to minimize such risks. Despite this challenge, patients can still achieve positive oncological outcomes with standard adjuvant therapy after surgery.

## Data Availability

The datasets used and/or analyzed during the current study are available from the corresponding author on reasonable request.

## Abbreviations

CST: Corticospinal tract
DES: Direct electrical stimulation
DTI: Diffusion tensor imaging
EOR: Extent of resection
GTR: Gross-total resection
HGGs: High-grade gliomas
LGGs: Low-grade gliomas
OS: Over survival
PFS: Progression-free survival
PR: Partial resection
STR: Subtotal resection
